# Methanolic Extract of Winter Cherry Causes Morpho-Histological and Immunological Ailments in Mulberry Pyralid *Glyphodes pyloalis*

**DOI:** 10.3389/fphys.2020.00908

**Published:** 2020-08-07

**Authors:** Zahra Afraze, Jalal J. Sendi, Azadeh Karimi-Malati, Arash Zibaee

**Affiliations:** ^1^Department of Plant Protection, Faculty of Agricultural Sciences, University of Guilan, Rasht, Iran; ^2^Department of Silk Research, Faculty of Agricultural Sciences, University of Guilan, Rasht, Iran

**Keywords:** panirbad, larval-pupal intermediates, detoxfying enzymes, hemocytes, midgut histology, ovarial malfunction

## Abstract

The effect of Withania somnifera a medicinal plant seed extract was tested against lesser mulberry pyralid, a potential pest of mulberry. The mulberry leaves were used for silk production in rural areas of northern Iran. The extract was administered orally by leaf dipping method in two lower (5%W/V) and higher (15%W/V) dosages to third instar larvae (<24 h) for biological assays and to fifth instar larvae (<24 h) for Physiological studies. The results showed formation of larvoids (Ls), larval-pupal intermediates (LPIs), pupoids (Ps) and pupal-adult intermediates (PAIs). The results showed increased larval duration by 1.7 and 2 folds in 5 and 15% treatment, respectively. Fecundity of resultant adults was decreased by 1.2 and 1.3 in 5 and 15% treatment, respectively. Except approximate digestibility (AD) and consumption index (CI) all other feeding indices showed reduction. The feeding deterrence was prominent at 15% (87%) and 5% showing 48% deterrence. Our enzymatic and non-enzymatic assessments upon treatment showed reduction in key components, except detoxifying enzymes. However, the activity of an important enzyme involved in cuticle hardening and immunity called phenoloxidase was reduced. We also investigated the histology of midgut for further analysis and found drastic changes in main cellular elements. Immunological changes following treatment was noticeable in reduced Total Hemocyte Count but surprisingly increased Differential Hemocyte Count. However, the hemocytes structure was extremely damaged. The reduced number of eggs in treated but survived adults indicated reduced ovaries, with vacuolization both in trophocytes and oocytes. The key chemical compounds showed reductions particularly at 15%. The present results are concomitant with few earlier studies on this medicinal plant and deserve further studies particularly in deriving key chemicals that alter metamorphosis similar to insect growth regulators.

## Introduction

The chemical plant protection agent usage has turned into an environmental disaster reverting the biological sustainability and ecological order. Thus, chemical pesticides are considered a threat to useful fauna, contaminates agricultural products and human health (Nicolopoulou-Stamati et al., 2016; Carvalho, 2017). That is why scientists, particularity environmentalists are in favor of non-chemical methods, that are safer for human, animals and the whole environment (Liao et al., 2017; Kunbhar et al., 2018). The chemical pesticides need to be substituted by natural ones, and in this context plants could play a major role (Isman, 2000; Govindarajan et al., 2016). Plant extracts and essential oils have a wide range of actions including repellents, attractants, anti-feedants, being toxic to larvae and eggs or retard insect growth by disrupting hormonal balance (Sarwar and Salman, 2015; El-Sheikh et al., 2016; Silva et al., 2018).

Winter cherry, *W. somnifera* L (Solanales: Solanaceae) is a local plant to east Mediterranean and south Asia (Parwar and Tarafdar, 2006) and has been used in traditional medicine (Bhattacharya et al., 2001). In Iran, this plant is better known locally as Panirbaad, grown only in Sistan and Baluchistan province, Khash (28°13′16″N 61°12′57″E) and Saravan (27°22′15″N 62°20′03″E) cities (Keykha et al., 2017). Antioxidant, anti-tumor, anti-inflammatory, anti-depressant, anti-anxiety, controlling blood sugar and this also effective on neural transmitters (Alam et al., 2012). Roghani et al. (2006) reported that this plant is useful for Parkinson disease. The major constituents that provide the winter cherry with the privilege in disease treatments, include withanine, withasomnine, somniferine, withaferins, withanolides, stigmasterol, and sitoinosides (Dar et al., 2015). Among the withanolides in this plant, withaferins A causes its inhibitory effect on cells and tumors is of great value in the pharmaceutical industry (Rai et al., 2016). Maheswari et al. (2020). Reported that Titanium dioxide (TiO_2_) nanoparticles modified with *W. somnifera* and *Eclipta prostrate* root extract had more anti-cancer activity than other biologically modified samples. Studies have shown that the *W. somnifera* seed contains fatty acids such as linoleic acid, oleic acid, palmitic acids, stearic acid, 11,14,17-eicosatrienoic acid, and nervonic acid, which can have a significant effect on the treatment of psoriasis-like skin etiologies (Balkrishna et al., 2020).

*Glyphodes pyloalis* Walker (Lep: Pyralidae) feed only on mulberry and it causes severe damage to the foliage. The larvae of this pest upon rolling the leaves eat the parenchyma, leaving only the veins. The insects in addition to direct loss are also responsible for indirect loss through transmission of viruses that are pathogenic to silk worm (Watanabe et al., 1988; Matsuyama et al., 1991). The winter cherry uses the withanoloid in the seeds as an anti-feeding and repellent against insects (Glotter, 1991). In addition psoralen and isopsoralen present in this seed plant act as anti-feedant and insecticide (Panwar et al., 2009). Various concentrations of winter cherry extract have resulted into mortality of adult rice weevil (Suvanthini et al., 2012). The growth inhibitory of seeds and roots of this plant has been reported on certain polyphagous pests, leading to inhibition of pupal and adult formation that effects of seeds were more severe than the roots of *W. somnifera* (Gaur and Kumar, 2017). The same authors also reported increased larval, pupal and adult duration after incorporation of root extract. Morphological and various growth disorders have been recorded in *Spodoptra litura* Fab (Lep: Noctuidae) (Gaur and Kumar, 2019). The prepupal treatment by root and seed extracts on *S. litura* and *Peicalliaa ricini* Fab (Lep: Arcttidae) showed that the seed extract exhibited mortality but not the root extract (Gaur and Kumar, 2020). Winter cherry extracts (aqueous suspension, ether and water) of roots, stems, leaves and fruits have been used against *Callosobruchus chinensis* L (Coleoptera: Chrysomelidae) adults where a 63.33% mortality have been observed using 10% ether extracts of its roots (Gupta and Srivastava, 2008). There have been certain work on mulberry pyralid using plant essential oils or extracts. The notable works which were investigated in our laboratory are as follows; the extract of medicinal plant known as sweet wormwood has shown deterrence, growth inhibition and affecting digestion. The energy reserves were also reduced compared to the control (Khosravi et al., 2011). Similarly, treatment of *G. pyloalis* larvae by a commercial product of neem (CIR-23, 925/96, 0.03% azadirachtin, India) showed significant anti-feedant activity, reduced nutritional indices. The effect was also profound in digestive enzymatic and energy reserves of this pest (Khosravi and Jalali Sendi, 2013). The mortality and the sublethal effect of *Thymus vulgaris* L. and *Origanum vulgare* L. essential on *G. pyloalis* Walker has been reported with the effects on some important enzymes (Yazdani et al., 2014). Similar activities were also found after treatment of *G. pyloalis* larvae with lavander essential oil (Yazdani et al., 2013). In order to have an understanding of insect behavior and physiology in response to their respective hosts, a basic knowledge of insect feeding indices are a prerequisite (Najar-Rodriguez et al., 2010).

Plants produce a set of chemicals based on their needs; those of nutritional values and the second group is the chemicals that are considered as secondary metabolites meant for the purpose of defense against invaders (War et al., 2013). During the course of evolution, plants have adopted themselves with chemicals they produce (Kabir et al., 2013; Isman and Grieneisen, 2014; Rajapakse et al., 2016). As far as insect herbivory is concerned, the plant may act as anti-feedants, repellents, toxicants, growth regulators and may even alter their immunological strategies (Murillo et al., 2014; Selin-Rani et al., 2016). There are several reports on anti-feedants by plant products against insects (Ragesh et al., 2016; Ali et al., 2017; De Santana Souza et al., 2018; Fite et al., 2018; Kaur et al., 2019). Plant products also act as repellents (Abtew et al., 2015; Camara et al., 2015; Nasr et al., 2015; Niroumand et al., 2016) growth regulatory such as malformed larvae, pupae and adult (Gnanamani and Dhanasekaran, 2013; Jeyasankar et al., 2014; Vasantha-Srinivasan et al., 2016).

Insects defend themselves by two methods against foreign bodies, a general method (i.e., cuticle and chemicals involve in defending insects) and the second one involving cellular and humeral responses (Beckage, 2011; Dubovskiy et al., 2016; Rahimi et al., 2019). The plant products are capable of indulging in both of them and thus weakening the insects and make them susceptible to various diseases and parasites (Veyrat et al., 2016; Gasmi et al., 2019).

There has been rapid growth in botanicals research in the past 20 years, however commercialization of new botanical are lagging behind. The pyrethrum and neem (azadirachtin) are still the standard class botanical pesticides that are in use in many regions of the world. Botanical products may have the greatest impact in developing countries especially tropical regions where the results of available and chemical insecticides use expensive and are a treat to consumers (Isman, 2019). Therefore, the research for safer and cheaper methods of control via available resource with minimal side effects is most welcome in developing countries.

## Materials and Methods

### Insect Culture

The mulberry pyralid larval stages were handpicked the mulberry plantations in Rasht district (37°17′ N, 49°35′ E) northern Iran. The larvae were reared on tender mulberry leaves (shin ichinoise variety) in a rearing chamber (set at 24 ± 1°C, 75 ± 5% relative humidity and 16:8 light:dark) in clear plastic jars (18 × 15 × 7 cm) with muslin cloth devised on the lid for aeration. The paired adults were released in pairing boxes. In order to feed the adults, a piece of cotton saturated with 10% honey solution was provided and for adults to lay eggs, mulberry leaf was provided. The leaves and the cotton wool soaked honey solution were changed daily.

### Methanolic Extraction of *W. somnifera* Seeds

Fresh fruits of winter cherry were procured from Saravan district (27°22′15″N 62°20′03″E) Sistan and Baluchistan province south east of Iran. The seeds were dried at room temperature initially and then in the oven at 50°C for 48 h. They were grounded by an electric grinder. Dried seed powder of winter cherry (30 g) was added to 300 ml of methanol 85% (Merck, Germany) and then stirred for 1 h on a stirrer. The resultant solution was transferred to 4°C for 48 h and then stirred for additional hour. Then solution was filtered through Whatman filter paper (No.4) and rotary evaporated and a black residue yielded. The residue was dissolved in 10 ml methanol and used as stock (Warthen et al., 1984). Dilutions were made with distilled water using 0.1% Triton X-100 (Darmstadt Germany).

### Development

Based on pretrial, two concentrations (i.e., 5 and 15%) were chosen and then used for bioassays. Circular leaf discs (6 cm in width) were drenched in related concentrations for 10 s. Control received methanol treated leaf discs in the same manner. For each treatment and control third instar larvae <24 h were used. This test was performed in 3 replications and each replication with 10 larvae. They were let to feed on treated food for 24 h after that, fresh leaves were provided to them. The insects were maintained at 25 ± 2°C, 65 ± 5% RH, and 14:10 light: dark in an incubator. The duration of various stages was monitored and recorded until all control adults died. The number of eggs from treated and control emerged adults were counted daily.

### Nutrition

To study nutritional values for treated and control insects the method of Waldbauer (1968) were adopted. For this purpose <24 h fifth instar larvae were maintained on treated and control diets for 3 days. The formulae were used; Approximate digestibility (AD) = 100 (E–F)/F, Efficiency of conversion of ingested food (ECI) = 100P/E, Efficiency of digested food (ECD) = 100 P/ (E–F), Consumption index (CI) = E/TA and Relative growth rate (RGR) = P/TA. A is the average of dry weight of larvae during the experiment, E is the dry weight of consumed food, F is the dry weight of produced feces, P the dry weight of the biomass of larvae and T is the duration of the experiment (4 days).

### Anti-feedant Bioassay

This method was adopted from Isman et al. (1990). In this method which was based on choice tests, The 10 fifth instar larvae were fed for 24 h on leaf disks control and treated. After the end of experiment the leaf discs were removed and were analyzed by device (leaf-area-meter A3 Light box UK). The feeding deterrence index was calculated with the formula as follows: FDI = (C-T)/(C+T) × 100. Here, C is the amount of leaf eaten in untreated (control) leaves and T is the amount of leaf eaten by of insects on treated leaves (Isman et al., 1990).

### Biochemical Assays

In order to estimate the amount of energy reserves like triglyceride, protein and glycogen, the whole body of treated fifth instar larvae and controls were first homogenized in an eppendorf vial with the help of a hand homogenizer in universal buffer (i.e., 50 mM sodium phosphate-borate at pH 7.1) and the supernatant was freezed at -20°C until use. The procedure of Lowry et al. (1951) was implemented to measure the total protein. To 100 μL of which reagent, 20 μL of supernatant was added, and then the incubation was done for 30 min at 25°C. The absorbance was recorded at 545 nm.

The amount of triglyceride was measured using the assay kit of Pars Azmoon, Tehran, Iran. The solution included a buffer (50 mM, pH 7.2), the chlorophenol (C_6_H_5_ClO 4 mM), 2 mM adenosine triphosphate (C_10_H_16_N_5_O_13_P_3_), 15 mM Mg^2+^, 0.4 kU/l glycerokinase, 2 kU/l peroxidase, 2 kU/l lipoprotein lipase, 0.5 mM 4-aminoantipyrine, and 0.5 kU/L glycerol-3-phosphate-oxidase. Ten microliter of the sample was incubated with 10 μL of distilled water and 70 μL of reagent for 20 min at 25°C. The formulae see below was used to estimate triglyceride amount:

mg/dL = optical density of the sample/optical density of standard × 0.01126.

The reading was done at 545 nm (Fossati and Prencipe, 1982) in an ELISA reader (Awareness, United States). For measuring the glycogen, the entire larvae were drenched into 1 mL of 30% KOH. The sample tubes covered with aluminum foil and boiled for 30 min. The tubes were first vortexed and then placed on ice cubes. A 2 mL 95% ethanol was used in order to separate the glycogen from the solution. Samples were vortexed again and left on ice bag for 30 min. The centrifugation was performed at 13,000 g for 30 min. The pellets of glycogen thus formed were collected and mixed in distilled water (1 mL) and then vortexed. Samples of standard for glycogen were prepared in ascending order (0, 25, 50, 75, and 100 mg/mL) and then mixed with phenol (5%). The samples were incubated on ice bath for 30 min. Finally, the absorbance was read at 490 nm (Chun and Yin, 1998).

The activity of α-amylase was estimated based on the method of Bernfeld (1955). The starch (1%) was used as substrate. The enzyme (10 μL) was incubated with Tris-HCl buffer (50 μL of 20mM at pH 7.1) and 20 μL of starch (1%) at 30°C for 30 min. After addition of 100 μL Dinitrosalicylic acid (DNS) it was heated in water tab set at boiling point for 1 minute and then read at 540 nm.

The method of Garcia-Carreno and Haard (1993) was used for determination of general proteases using 1% azocasein as a substrate for their activity. For this purpose, supernatant (10 μL) and buffer (15 μL) and 50 μL of substrate were incubated for 3 h at 37°C in an oven. In order to stop the reaction 150 μL of 10% trichloroacetic acid was added. The blanks were prepared by addition of trichloroacetic acid to the substrate. The liquids were then kept in a refrigerator (4°C) for 30 min, and then centrifugation was done at 13,000 g. Later, mixture of supernatant and NaOH, 100 μL each were transferred to ELISA plates and read at 440 nm.

The activity of lipase enzyme was measured according to the method of Tsujita et al. (1989). Using 18 μL of the substrate p-nitrophenyl butyrate (50 mM) and then mixing it with midgut extract (10 μL) to which 172 μL of universal buffer solution (1 M) (pH 7) was included and then incubated at 37°C and read at 405 nm.

For estimation of α-glucosidase and β-glucosidase the method used by Silva and Terra (1995) was followed where 15 μL of the enzyme solution was incubated with 30 μL of p-nitrophenyl-α- glucopyranoside (5mM) a substrate for α-glucosidaseand p-nitrophenyl-β-glucopyranoside (5mM) as substrate for β-glucosidase, respectively. Then, to each of the solutions 50 μL of universal buffer (50 mM sodium phosphate-borate pH 7.1) added and allowed to react for 10 min at 37°C. Followed by observation at 405 nm.

The general esterases activity followed the methods of Van-Asperen (1962). One whole gut was first homogenized in 1000 μL of 0.1 mM phosphate buffer (pH 7) which included Triton x-100 (0.01%), and solution was centrifuged at 10,000 g for 10 min at 4°C. The microtubes were replaced with new microtubes phosphate buffer was added to each and observation was done at 630 nm.

In order to determine glutathione s- transferase (GST) activity, the procedure of Habing et al. (1974) was incorporated using 1-chloro-2, 4-dinitrobenzene (CDNB) (20 mM) as the substrate. The homogenized larva with 20 μL distilled water was centrifuged at 12,500 g for 10 min at 4°C. Fifteen microliter of supernatant and 135 μL phosphate buffer (pH 7) with 50 μL of CDNB were mixed with 100 μL of GST. Change of absorbance at 340 nm was recorded for 1 min in 9 s intervals at 27°C.

### Phenoloxidase Activity Assay

For measuring phenoloxidase activity, hemolymph and ice-cold sterile phosphate buffer saline (PBS) was used in a ratio of 10–90 μL, respectively. The L-DOPA (3, 4- dihydroxyphenylalanine) (10 mM, Sigma-Aldrich Co., United States) was used as the substrate for assaying this enzyme following the procedure of Catalán et al. (2012), with some modifications. Centrifugation of the samples was performed at 5,000 g (4°C and 5 min). Then 50 μL of solution was mixed with 150 μL of the amino acid L-DOPA. The activity was calculated by division of absorbance with the amount of protein in hemolymph. However, the protein content was measured following the method of Lowry et al. (1951). The specific activity of phenoloxidase was recorded at 490 nm during the reaction.

### Histology of Larval Midgut and Adult Ovary

The digestive system of *G. pyloalis* fifth instar larvae after being fed on treated leaves from third instar onwards were dissected out under a stereomicroscope (Olympus Japan) in isotonic ringer saline. The dissected digestive system were immediately fixed in Buin’s fluid for 24 h. Then washed first in tap water and then distilled water. They were processed for dehydration in ethanol grades (30, 50, 70, 90% and then absolute), and the paraffin was used for embedding. The sections were cut at 5 μm thickness by a rotary microtome (Model 2030; Leica, Germany). Routine staining by hematoxylin and eosin (Merck) was used. Photos in control vs. treatments were taken whenever necessary under a light microscope (M1000 light microscope; Leica) equipped with an EOS 600D digital camera (Canon, Japan). The ovary of 2 days old adults were dissected in the similar way and processed as described for gut. However, the sections were cut longitudinally.

### Total and Differential Hemocyte Count (THC and DHC)

After 48 h, the hemolymph of fifth instar larvae (in two concentrations used and controls) were collected from the first abdominal pro leg. For THC a Neubauer hemocytometer (HBG, Germany) was used. For this purpose, the larval hemolymph (10 μL) was mixed with 290 μL of anti-coagulant solution (0.017 M EDTA, 0.041 M Citric acid, 0.098 M NaOH, 0.186 M NaCl, pH 4.5) (Amaral et al., 2010). The DHC was counted by immersing the larvae in a hot distilled water (60°C) for 5 min, after drying with blotting paper, the first abdominal pro leg was excised and a drop of hemolymph was released on to a clean slide and a smear was made using another slide. The air-dried smears were stained with 1:10 diluted stock Giemsa (Merck, Germany) for 14 min, then were washed in distilled water. The smears were dipped for 5 s in saturated lithium carbonate (LiCO_3_) for differentiation of cytoplasm and nucleus and then washed again in distilled water for a few minutes. They were dried at room temperature and then permanent slide was prepared in Canada balsam (Merck, Germany). The cells were identified based on the morphological characteristics observed under a microscope (Leica light-microscope) (Rosenberger and Jones, 1960). Two hundred cells were randomly counted from four corners and a central part of each slide (Wu et al., 2016). Totally, 800 cells of four larvae were counted and the percentage of each cell type was estimated. The number of cells in controls was also simultaneously recorded.

### Phase Contrast Microscopy

The hemolymph was collected from each incised larval proleg and was immediately mixed with anti-coagulant (0.186 M NaCl, 0.098 M NaOH, 0.041 M citric acid, and 0.017 M EDTA, pH 4.5). Five microliter of the solution was placed over a glass slide making a thin film using cover glass. The various hemocytes were identified by phase contrast microscopy, and photos were taken using in built camera microscope.

### Statistical Analysis

All the data in relation to larva, pupal and adult duration were analyzed by one-way ANOVA (SAS Institute, 1997). Similarly, the data collected from non-enzymatic and enzymatic assays were also analyzed in the same way. All the means were separated using Tukey’s multiple comparison test (*p* < 0.05).

## Results

### Effect of Methanolic *W. somnifera* Extract on Larval-Pupal-Adult and Fecundity of *G. pyloalis*

Feeding 3rd instar larvae with treated leaves of *W. somnifera* extract resulted in various deformities and lengthening durations (Table 1). As it is depicted in the 5 and 15% extracts significantly prolonged larval duration (17.99 ± 0.74 and 21.93 ± 1.23 days) compared to control (11.26 ± 0.38 days), respectively. Prepupal duration was only significant at 15% extract compared to 5% and the control (*F* = 3.70; *df* = 2, 44; *p* = 0.03). In case of pupae, the longest duration was observed at 15% compared to 5% and control (*F* = 15.67; *df* = 2, 44; *p* = 0.0001). Adult duration was longest in control male and female and being shortest with 15% treatment (*F* = 8.71; *df* = 2, 14; *p* = 0.0046) and (*F* = 26.60; *df* = 2, 14; *p* = 0.0001), respectively. The 5% treatment shortened only adult female longevity compared to control. Fecundity was highest in control and lowest at 5 and 15% (*F* = 228.69; *df* = 2, 14; *P* = 0.0001).

**TABLE 1:** Effect of *Withania somnifera* methanolic extract on *Glyphodes pyloalis* developmental stages.

**Treatments**	**Larval duration (days)**				**Prepupa (days)**	**Pupa (days)**	**Longevity (♂)**	**Longevity (♀)**	**Fecundity (No.)**
	**3rd instar**	**4th instar**	**5th instar**	**Total**					
Control	2.40 ± 0.13b	3.33 ± 0.12c	5.53 ± 0.13c	11.26 ± 0.38c	2.33 ± 0.12b	8.20 ± 0.17c	3.40 ± 0.24a	4.6 ± 0.24a	101 ± 2.25a
T5*	4.66 ± 0.12a	5.33 ± 0.43b	8 ± 0.19b	17.99 ± 0.74b	2.6 ± 0.13ab	9.40 ± 0.32b	2.40 ± 0.4ab	2.8 ± 0.2b	50.6 ± 1.72b
T15	5.20 ± 0.2a	7.13 ± 0.52a	9.60 ± 0.51a	21.93 ± 1.23a	2.8 ± 0.10a	10.86 ± 0.45a	1.06 ± 0.24b	2 ± 0.31c	32.1 ± 2.93c

*T5 = 5% and T15 = 15%. *Means in a column followed by the same letters are not significantly different (p < 0.05 Tukey’s test).*

### Nutrition

The nutritional indices including AD, ECD, ECI, RGR, and CI and FDI were calculated both in control and treatments (Table 2). The highest AD was observed at 15% treatment (*F* = 6.95; *df* = 2, 8; *p* = 0.02) and the lowest in control (56.97 ± 10.46). The highest ECD and ECI belonged to controls 9.12 ± 5.29% and 8.99 ± 1.05% (*F* = 7.52; *df* = 2, 8; *p* = 0.02 and (*F* = 11.99; *df* = 2, 8; *p* = 0.008). Negative index was recorded for ECD and ECI at 5 and 15% treatment -4.90 ± 1.65% and -7.84 ± 1.42% and -20 ± 8.37% and -198.41 ± 55.5% (*F* = 7.52; *df* = 2, 8; *p* = 0.02) and (*F* = 11.99; *df* = 2, 8; *p* = 0.008), respectively. Our results demonstrated the highest CI value at 5% and lowest at 15% and control (*F* = 0.49; *df* = 2, 8; *p* = 0.6348). Relative growth rate (RGR) was significantly decreased at 15% treatment (*F* = 30.74; *df* = 2, 8; *p* = 0.0007). The feeding deterrence index of *W. somnifera* seed extract was calculated for 24 h. As it is shown in Table 2, the increase in concentrations caused increase in percent deterrence. The highest concentration 15% used the deterrence was recorded 87.7 ± 0.98 (*F* = 63.65; *df* = 2, 8; *p* = 0.0001).

**TABLE 2:** Nutritional indices in fifth-instar larvae of *Glyphodes pyloalis* feeding on two concentrations of *Wihania somnifera.*

**Treatment**	**AD (%)**	**ECD (%)**	**ECI (%)**	**RGR (mg/mg day^–1^)**	**CI (mg/mg day^–1^)**	**(FDI) (%)**
Control	56.97 ± 10.46b	9.12 ± 5.29a	8.99 ± 1.05a	0.10 ± 0.01a	2.95 ± 0.53a	0c
T5*	76.67 ± 7.04ab	-4.90 ± 1.65ab	-20 ± 8.37a	-0.13 ± 0.04b	3.67 ± 0.86a	48.13 ± 6.58b
T15	95.59 ± 1.34a	-7.84 ± 1.42b	-198.41 ± 55.55b	-0.2 ± 1.96b	2.83 ± 0.46a	87.7 ± 0.98a

**^∗^T5 = 5% and T15 = 15% Means with the same lowercase letters in a column are not significantly different at P < 0.05 (Tukey’s test). AD, approximate digestibility; ECI, efficiency of conversion of ingested food; ECD, efficiency of conversion of digested food; CI, consumption index; RGR, relative growth rate; FDI, feeding deterrence index.**

### Enzymatic Assay

The results demonstrated that, treatment of larvae with *W. somnifera* seed extract caused a significant decrease in α-amylase activity after 48 h (*F* = 49.44; *df* = 2, 8; *P* = 0.0002). Similarly, the activity of α- and β-glycosidase was reduced significantly with 15% treatment after 48 and 72 h (0.046 ± 0.0026 and 0.029 ± 0.0052, respectively). At 5% treatment there was no change after 48 h but a reduction was followed 72 h later (0.061 ± 0.0102 and 0.066 ± 0.0053). The activity of lipase was also inhibited by 5 and 15% about 1.5 and 7 fold, respectively. The same trend was also followed in general proteases (Table 3).

**TABLE 3:** Digestive enzyme activities in fifth instar larvae of *Glyphodes pyloalis* after treatment with two concentrations of *Withania somnifera* extract.

**Digestive enzymes**	Time*	**Control**	**T5^†^**	**T15^†⁣†^**	***F***	***P***	***df***
α-amylase (U/mg protein)	48; 72	0.157 ± 0.0047a; 0.189 ± 0.0040a	0.113 ± 0.0082b; 0.135 ± 0.0074b	0.076 ± 0.0029c; 0.118 ± 0.0026b	49.44; 52.63	0.0002; 0.0002	2, 8; 2, 8
α-glucosidase (U/mg protein)	48; 72	0.070 ± 0.0058a; 0.080 ± 0.0047a	0.033 ± 0.0059b; 0.058 ± 0.0038b	0.046 ± 0.0026b; 0.029 ± 0.0052c	13.95; 30.30	0.0055; 0.0007	2, 8; 2, 8
β-glucosidase (U/mg protein)	48; 72	0.063 ± 0.0072a; 0.085 ± 0.0037a	0.061 ± 0.0102a; 0.066 ± 0.0053ab	0.037 ± 0.0057a; 0.050 ± 0.0079b	3.17; 8.70	0.1152; 0.0168	2, 8; 2, 8
Lipase (U/mg protein)	48; 72	0.322 ± 0.0099a; 0.425 ± 0.0303a	0.368 ± 0.0135a; 0.335 ± 0.0201ab	0.297 ± 0.0507b; 0.066 ± 0.0382b	1.34; 12.55	0.3307; 0.0072	2, 8; 2, 8
Protease (U/mg protein)	48; 72	0.065 ± 0.0057a; 0.090 ± 0.0093a	0.035 ± 0.0029b; 0.031 ± 0.0027b	0.021 ± 0.0059b; 0.004 ± 0.0016c	19.24; 59.13	0.0025; 0.0001	2, 8; 2, 8

*48 and 72 h after treatments ^†,††^ 5 and 15% treatments, respectively. Means (±SE) followed by the same letters in a row indicate no significant difference (P < 0.05) according to the Tukey’s multiple comparison test.

### Detoxifying Enzymes

The detoxifying enzymes including glutathione-*S*-transferase, α-naphtyl acetate and β-naphtyl acetate substrates were analyzed after treatment with 5 and 15% concentrations of *W. somnifera* seed extract which are depicted in Table 4. The overall results showed increase in these parameters (Table 4). However, the phenoloxidae activity decreased at 5 and 15% treatment after 24 and 48 h (Table 4).

**TABLE 4:** Detoxifying enzyme activities in fifth instar larvae of *Glyphodes pyloalis* after treatment with *Withania somnifera* methanolic extract.

**Detoxifying enzymes**	Time*	**Control**	**T5^†^**	**T15^†⁣†^**	***F***	***P***	***df***
Glutathione S-transferase; (U/mg protein)	48; 72	0.020 ± 0.0033b; 0.025 ± 0.0030c	0.087 ± 0.010a; 0.127 ± 0.0086b	0.110 ± 0.0060a; 0.164 ± 0.0063a	43.27; 125.21	0.0003; 0.0001	2, 8; 2, 8
α-naphtyl acetate; (U/mg protein)	48; 72	0.041 ± 0.0039c; 0.124 ± 0.0076b	0.081 ± 0.0040b; 0.126 ± 0.0024b	0.127 ± 0.010a; 0.170 ± 0.0069a	38.7; 17.88	0.0004; 0.003	2, 8; 2, 8
β-naphtyl acetate; (U/mg protein)	48; 72	0.048 ± 0.0037a; 0.072 ± 0.0056b	0.060 ± 0.0057a; 0.104 ± 0.0039a	0.062 ± 0.0062a; 0.125 ± 0.0074a	2.05; 20.73	0.2096; 0.002	2, 8; 2, 8
Phenoloxidase; (U/mg protein)	48; 72	0.380 ± 0.0109a; 0.466 ± 0.0096a	0.259 ± 0.0070b; 0.139 ± 0.0069b	0.234 ± 0.0105b; 0.118 ± 0.0080c	69.52; 574.25	0.0001; 0.0001	2, 8; 2, 8

*48 and 72 h after treatments ^†,††^ 5 and 15% treatments, respectively. Means (±SE) followed by the same letters in a row indicate no significant difference (P < 0.05) according to the according to the Tukey’s multiple comparison test.

### Energy Reserves

The amount of energy reserves like (protein, triglyceride) were decreased significantly in both concentrations after 48 and 72 h. While at 5% concentration the amount of glycogen decreased 1.5-fold and that of triglyceride 1.8-fold (Table 5).

**TABLE 5:** Energy reserves of fifth instar *Glyphodes pyloalis* larvae after treatment with methanolic extract of *Withania somnifera*.

**Macromolecules**	Time*	**Control**	**T5^†^**	**T15^†⁣†^**	***F***	***P***	***df***
Total protein (mg/dl)	48; 72	1.142 ± 0.0186a; 1.116 ± 0.0277a	1.065 ± 0.0198b; 0.980 ± 0.0216b	0.998 ± 0.0129b; 0.801 ± 0.0332c	17.28; 31.83	0.0032; 0.0006	2, 8; 2, 8
Glycogen (mg/dl)	48; 72	0.035 ± 0.0007a; 0.031 ± 0.0014a	0.029 ± 0.0006b; 0.028 ± 0.0004a	0.025 ± 0.0006c; 0.019 ± 0.0017b	52.13; 21	0.0002; 0.002	2, 8; 2, 8
Triglyceride (mg/dl)	48; 72	0.0131 ± 0.0003a; 0.0130 ± 0.0002a	0.0120 ± 0.0002ab; 0.0128 ± 0.0003a	0.0114 ± 0.0002; 0.0113 ± 0.0003b	9.87; 10.53	0.012; 0.0109	2, 8; 2, 8

*48 and 72 h after treatments ^†^, ^†⁣†^5 and 15% treatments, respectively. Means (±SE) followed by the same letters in a row indicate no significant difference (P < 0.05) according to the Tukey’s multiple comparison test.

### Histology

#### Midgut

**FIGURE 1:**
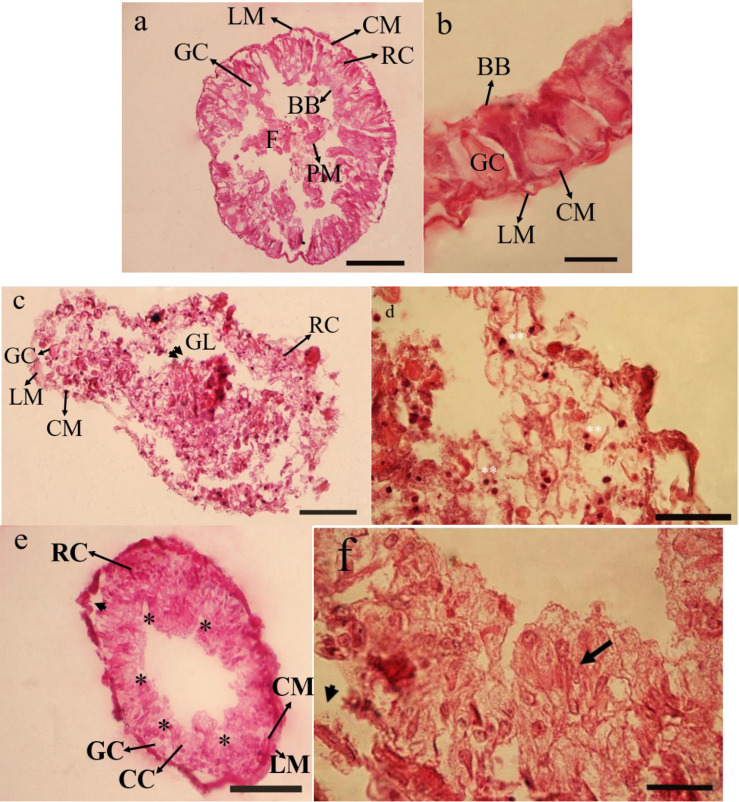
Effect of *Withania sotnnifera* seed extract on midgut histology of *Glyphodes pyloalis.* The midgut transverse section stained with hemotoxylin and eosin showing intact epithelial cells and pronounced goblet cells, regenerative cells, brush border and peritrophic membrane in controls **(a,b)**. The same in 5% treated larvae showing degenerative epithelium projecting into gut lumen (double arrow head) with the nucleus being extruded and highly stained (Double stars) **(c,d)**. **(e,f)** is depicting the same at 15% treatment with plenty of vacuolizations (*) separation of principal cells from basement membrane (Arrow head) and overcrowding of cells not recognizable into its respective names (Arrow). RC, Reganarative cell; GC, Goblet cell; LM, Longitudinal muscle; CM, Circular muscle; GL, Gut lumen; BB, Brush border; PM, Peritrophic membrane; F, Food.

The principal midgut cells in control is intact with large columnar cells and a prominent nucleus. The other prominent cells of midgut include goblet cells and regenerative cells. All the muscle layers are intact including longitudinal and circular ones. Peritrophic membrane and brush borders are also significantly clear (Figures 1a,b). In treatment 5% no clear differences between cells were observed and the midgut epithelium seemed to protrude inside gut epithelium giving it a dislodging surface. Nucleus were extruded from rupturing cells and densely stained in comparison to controls (Figures 1c,d). While in the treatment 15% a rupture of the cell membrane and some signs of necrosis in both nuclei and cytoplasm of the epithelial cells were observed. Large vacuoles were present in the epithelial layer of treated larvae. The principal cells were seen separating from their basement membrane. The overcrowding in the cellular structure making them not to be recognized into their counter parts in controls (Figures 1e,f).

### Immunological Effect

#### Total Hemocyte Count (THC)

**FIGURE 2:**
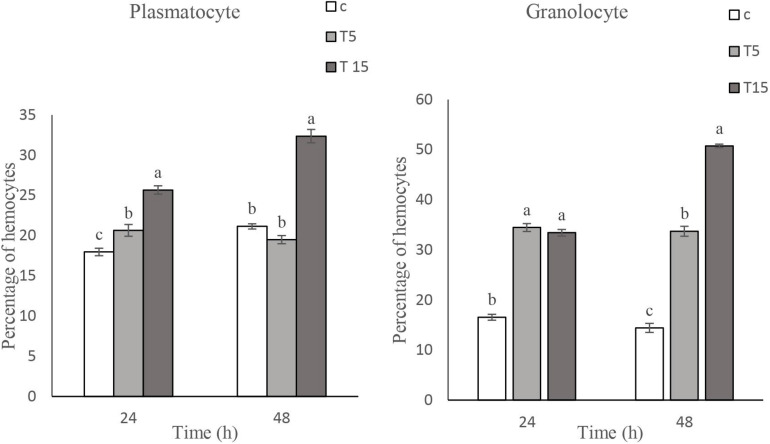
Mean percentage of plasmatocytes (PLs) and granulocytes (Grs) counts after treatment of fifth instar *Glyphodes pyloalis* larvae with 5% (T5) and 15% (T15) of *Withania somnifera* methanolic extract compared to control (C). Mean ± SE followed with the same letters above bars indicate no significant difference (*p* = 0.05). According to a Tukey’s test.

Effects of *W. somnifera* extract on total hemocyte count (THC) are shown in Figure 2. As depicted in Figure 2, the lowest THC are observed at 15% treatment after 24 h (*F* = 45.10, *df* = 2, 11; *p* < 0.0001). A significant decrease in THC was detected after 48 h (*F* = 21.57, *df* = 2, 11, *p* < 0.0004). However, no changes were observed at 5 and 15% treatment after 24 h.

#### DHC

**FIGURE 3:**
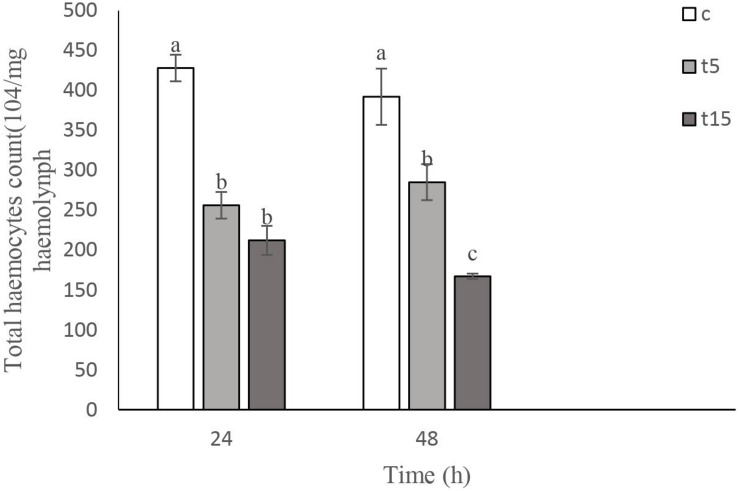
Total hemocyte count (THC) following treatment with 5 (T5) and 15% (T15) with *Withania somnifera* methaolic extract compared to control (C) in Vth instar larvae of *Glyphodes pyloalis* after 24 and 48 h). Mean ± SE followed with the same letters above bars indicate no significant difference (*p* = 0.05). According to a Tukey’s test.

The main cells after staining with Gimsa and phase contrast microscopy depicted 5 cells types so called Prohemocyte (Pr), Plasmotocyte (Pl), Granulocyte (Gr), Spherulocytes (Sph), and Oenocytoid (Oe) and a subtype of Plasmatocyte so called as Vermicytes. Treatment with 5% and 15% *W*. *somnifera* extract brought about changes in the number of PLs and GRs which are the main immunity involved cells (Figure 3). After 24 h of treatment with 5 and 15% of the extract the number of Pls increased significantly (*F* = 44.68; *df* = 2, 8; *P* < 0.0002). However, after 48 h there was no changes between control and 5% treatment. But in the 15% treatment the PLs number increased (*F* = 54.04; *df* = 2, 8; *P* < 0.0001). The number of GRs after treatment with 5 and 15% were increased after 24 h. The increase was significant in both the treatments compared with the controls, but no differences between the treatments (*F* = 153.80; *df* = 2, 8; *P* < 0.0001). The increase in the number GRs after 48 h was highly significant in the higher dosage used compared to control and the other treatment and 5% treatment was also significantly higher than the control but lower that 15% treatment (*F* = 212.89; *df* = 2, 8; *P* < 0.0001).

### Morphology of Hemocytes

**FIGURE 4:**
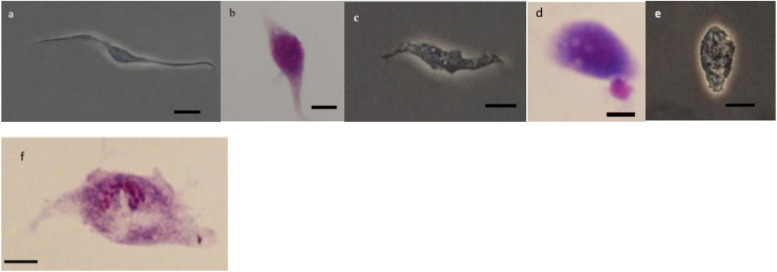
Effect of *Withania somnifera* seed extract on the morphology of plasmatocytes (PLs) in *Glyphodes pyloalis* in controls **(a,b)** and in treatments (**c,d**, 5%) and (**e,f**, 15%) (Bar = 10 μ).

**FIGURE 5:**
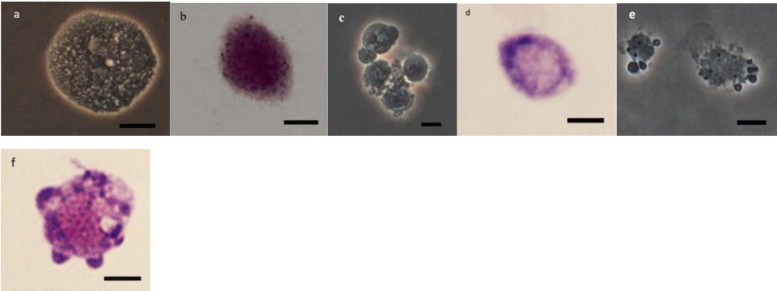
Effect of *Withania somnifera* seed extract on the morphology of granulocytes (GRs) in *Glyphodes pyloalis* (**a,b** control), (**c,d**, 5%) and (**e,f**, 15%) (Bar = 10 μ).

**FIGURE 6:**
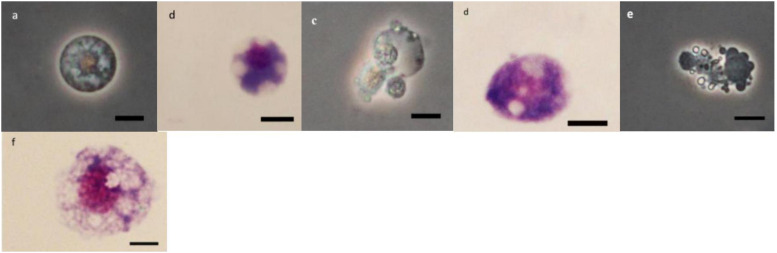
Effect of *Withania somnifera* seed extract on the morphology of spherulocytes (Sphs) in *Glyphodes pyloalis* (**a,b** control), (**c,d**, 5%) and (**e,f**, 15%) (Bar = 10 μ).

**FIGURE 7:**
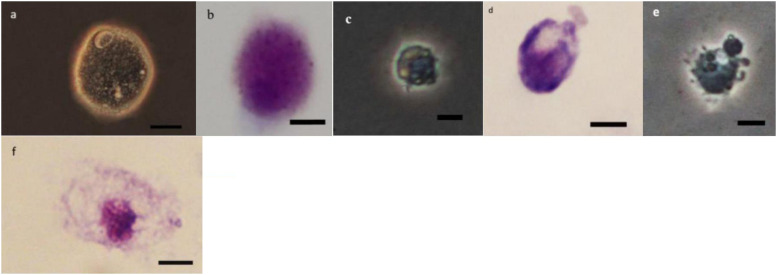
Effect of *Withania somnifera* seed extract on the morphology of oenocytoids (OEs) in *Glyphodes pyloalis* (**a,b** control), (**c,d** 5%), and (**e,f** 15%) (Bar = 10 μ).

**FIGURE 8:**
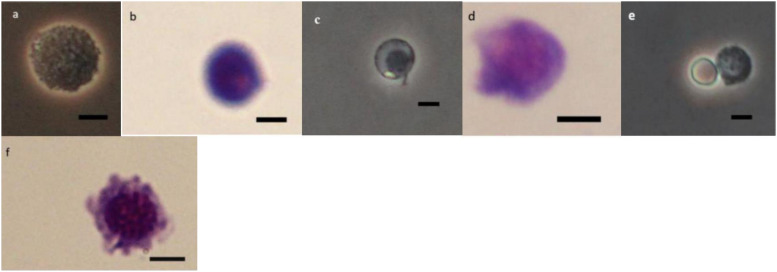
Effect of *Withania somnifera* seed extract on the morphology of Prohemocytes (PRs) in *Glyphodes pyloalis* (**a,b** control), (**c,d**, 5%) and (**e,f**, 15%) (Bar = 10 μ).

As it can be observed in Figures 4–8 drastic changes were seen in the morphology of studied hemocytes after treatment of 5th instar larvae with *W. somnifera* methanolic extract. The pleomorphic PLs in control were largely Vermicytes both in phase contrast Microcopy and Giemsa stained with typical long cytoplasmic extensions and a prominent nucleus (Figures 4a,b). However, in treatments, the changes seen are as follows; the cells showed lots of extrusions giving it an irregular shape and making it unidentifiable (5% treatment Figure 4c) showing vocalizations not normally observed in untreated cells (Figure 4d). The changes seen by 15% treatment are notable in the loss of cytoplasmic extensions, vacuolization and gradual degeneration (Figures 4e,f). The Gr in control were typically filled with plenty of granules and a prominent central nucleus (Figures 5a,b). However, in treatments the cells were degranulated and with lots of extruding materials giving the cells form of bulging appearances in both the treatments (Figures 5c–f). The normal Spherulocytes appear circular with a small but prominent nucleus and plenty of regular spherules (Figures 6a,b). After treatment the cells lost their integrity, became irregularly shaped, thus making the identification difficult (Figures 6c–f). The Oenocytoids, are rare cells but they are large with plenty of inclusions, the nucleus is typically smaller compared with cytoplasm and is located near the cell boundary (Figures 7a,b). However, in treated larvae these cells appeared vacuolated with no nuclear boundary and were irregularly shaped (Figures 7c,d at 5% treatment) and with extruded or disintegrating nucleus (Figures 7e,f). The Prohemocytes are small cells, the nucleus is prominent filling the cytoplasm (Figures 8a,b) but after treatment with the extract the cells were vacuolated and the nucleus were smaller compared to controls or the extruding bodies appearing at the surface showing the sign of disintegration (Figures 8c–f).

### Morphology, Histology and Energy Reserves of Ovaries

**FIGURE 9:**
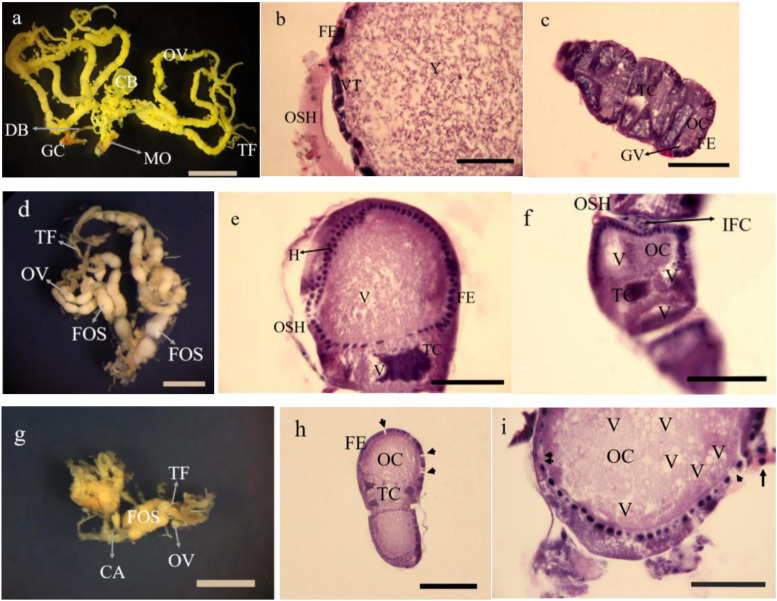
Gross morphology and histology of ovaries in *Glyphodes pyloalis* of 2 day old adults in controls (**a** Bar = 2 mm, **b,c** Bar = 60μ). The same in 5% treatments (**d** Bar = 2 mm, **e,f** Bar = 60μ) and 15% treatment (**g** Bar = 2 mm **h,i** Bar = 50μ). CB, Corpus Bursae; DB, Dactus Bursae; MO, Median Oviduct; GC, Genital Chamber; OV, Ovariole; TF, Terminal Filament; FE, Follicular Epithelium; VT, Vitellarium; OSH, Ovarial Sheath; Y, Yolk; TC, Trophocyte; OC, Oocyte; GV, Germinal Vesicle; FOS, Fused Oocytes; H, Hyperplasia; V, Vacuole; IFC, Inter Follicular Cells; CA, Calyx; Degenerating follicular epithelium (Single arrow head), Disintegrating follicular epithelium (Arrow) and Necrotic inter follicular epithelium (Double arrow head).

The dissected ovaries in treated insects were smaller than controls about 2 and 3 folds at 5 and 15% treatments, respectively. The number of oocytes were fewer than controls (Figures 9a–c in control, Figures 9d,g in 5 and 15% treatments). The histology in dissected ovaries of treated insects showed no sign of complete formation of oocytes in treatments compared to control (Figures 9b,c). The changes were mostly seen in the form of disintegration of epithelial cells without cell boundaries in treatments vacuolization both trophocytes and oocytes (Figures 9e,f,h,i) with no sign of vitellin and chorion formation with obvious oosorption seen in gross morphology.

**FIGURE 10:**
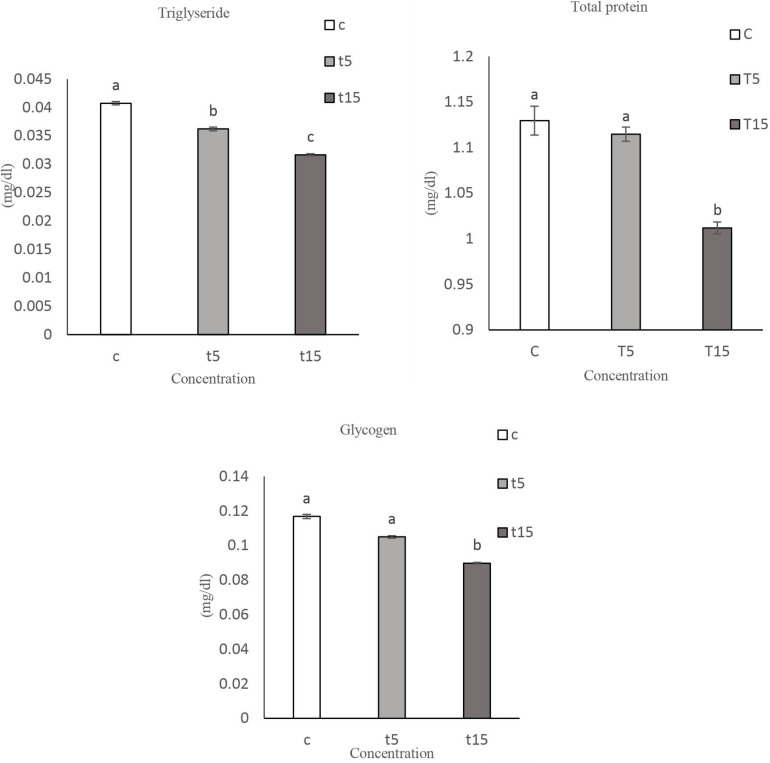
The amount of Protein, Triglyceride and Glycogen, in ovaries of 2 day old adult *Glyphodes pyloalis* in controls (C), in 5% treatment (T5) and in 15% treatment (T15). Mean ± SE followed with the same letters above bars indicate no significant difference (*p* = 0.05). According to a Tukey’s test.

The amount of total protein, glycogen and triglyceride were reduced by 15% in comparison to control and low dosage treatment (Figure 10).

## Discussion

The extract of various parts in *W. somnifera* has been reported as a potent insect growth regulator (Gaur and Kumar, 2017, 2018; Gaur and Kumar, 2019). The current study has focused on different effects of methanolic seed extract of this plant exerted on an important pest of mulberry. This pest is also suspicious of transmitting viral infection in silkworm. Treatment of seeds by this extract in two dosages selected with pretrials showed extensive growth inhibition. The growth inhibitory effect was prominent in the formation of intermediates larva-pupal-adults. In extreme cases we also noticed a 3th instar larva which could not shed its old cuticle. The morphological abnormalities might be related to chemicals in the extract that has already been reported as withanolids and withaferins (Trivedi et al., 2017; Gaur and Kumar, 2020).

Thus, we looked for possible reasons underlying these subtle circumstances upon treatment, therefore some aspects of nutrition in treated larvae were considered. The results indicated adverse impact of *Withania* extract on almost all measured nutritional indices. These adverse effects led to the various abnormalities, which are very well documented by various plant extracts including *Withania* (Senthil-Nathan, 2006, 2013; Shekari et al., 2008; Hasheminia et al., 2011; Khosravi et al., 2011). However, our results was interestingly different from other studies in giving negative ECD, ECI, and RGR which is indicative of its extreme anti-feedant activity. The feeding deterrence recorded 48 and 87% for 5 and 15% treatments, respectively, clearly indicated the anti-feedant activity (Qi et al., 2003; Luo et al., 2005; Junhirun et al., 2018). This anti-feedant effect is due to the presence of compounds as withanolides such as withanolide A and withanolide D in this plant therefore the insect is unable to efficiently convert food (Budhiraja et al., 2000). Digestion is a process of interactions taking place between chemicals (enzymes) produced by the same set of cells of midgut that absorb the nutrients. The activity of selected enzymes including, αamylase, α- and β-glycosidase, lipase and general proteases were affected and reduced particularly 72 h post treatment, which supports our previous results that the nutritional reductions follows the lower activity of enzymes (Rharrabe et al., 2008; Khosravi et al., 2010; Hasheminia et al., 2011) and other reports are also indicative in inhibition of important enzymes in their respective studies (Senthil-Nathan, 2013; Amin et al., 2019). George et al. (2018) reported the presence of a lectin in *Withania* leaves which showed insecticidal, growth inhibition and could also damage the secretory cells of midgut. The lectins or agglutinins with binding ability to specific carbohydrates are present in certain plant tissues as a defensive strategy against herbivores (Michiels et al., 2010). A fractioned lectin from *Polygonum persicaria* exhibited inhibitory activity of certain enzymes in *Helicoverpa armigera* leading to death of this insect (Rahimi et al., 2018).

Macromolecules, like proteins play a major role in organisms for development, growth and performing other vital activities (Chapman, 2013). In this study the reduced amount of protein could be attributed to insect inability to synthesize it or the synthesized protein is broken down to compensate the detoxification process (Vijayaraghavan et al., 2010). The Lipids and glycogens are stored reserves of insects (Chapman, 2013). As many insects are dependent for their adult stage reproductive process solely on the nutrients collected during their immature stages. Thus reduction of these key components are depicted in at least in two stages, first in the developmental stages showing various deformities or intermediates due to lack in energy reserves and second in adult stage where the longevity and egg production are severely affected. There are a number of proofs appearing in the literature for the shortage of critical macromolecules involved after the application of chemical stresses including plant products (Smirle et al., 1996; Etebari et al., 2007; Rharrabe et al., 2008; Shekari et al., 2008; Khosravi et al., 2010; Zibaee, 2011).

Detoxification is a phenomenon for reduction of the toxic effects of exogenous compounds that may be received by any organism (Amin et al., 2019). Two important detoxifying compounds are esterases and glutathione-s-transferases. Both enzymes were increased, particularly 72 h post treatment, certainly for removal of the exerted side effects of *Withania* toxicity. This result is similar to other reports of plant products used against insects (Kumrungsee et al., 2014; Murfadunnisa et al., 2019).

Phenoloxidase (PO) is an enzyme that is a link between cellular and humoral immunology of insects (Chapman, 2013). Usually when the insect is attacked by foreign bodies like fungal hyphae, nematodes or egg of parasitoids, this enzyme is released by insect oenocytoids for melanization of invaders to isolate them from damaging the insect tissues (Huang et al., 2002; Yu et al., 2003; Ling et al., 2005; Arakane et al., 2009; Beckage, 2011). The reports indicate decreased activity of PO by IGRs (Zibaee et al., 2012; Mirhaghparast and Zibaee, 2013; Rahimi et al., 2013). The *W. somnifera* has the characteristics of an IGR which is particularly observed in its ability to form incomplete metamorphosis, leading to various intermediate forms. Therefore, decrease in PO could be related to the IGR behavior of this compound.

The middle midgut is the main site of digestion and absorption in many insect orders (Selin-Rani et al., 2016). In our study, the damages to midgut cells that are responsible for secreting digestive enzymes were severe which clearly supports the inhibition of enzyme activity already reported and discussed above. The damages to midgut cells appears in two forms, one through binding of the extract (lectin) to carbohydrates and secondly damaging the tissues thereby, the enzymes are lowered or not produced at all. Lectin in the leaves of *W. somnifera* causes damage to midgut secretory cells have been reported recently (George et al., 2018) which corresponds with the present study. The reports by several workers that have tried other plant extracts or even essential oils against the gut of various insect pests are also indicative of a similar finding (Vasantha-Srinivasan et al., 2016; Murfadunnisa et al., 2019).

Cellular immunity of insect is considered as an important immunological system that provides to the insect to get rid of the intruders (Nation et al., 2008). This ability may take at least three forms, to phagocytize small intruders, form nodules orencapsulate larger intruders. The plant extracts exert their effects on cellular immunity by changes the THC and DHC. There are plenty of reports by various scientists, both in reducing or increasing cell numbers (Hassan et al., 2013). The reduced THC in the present study corresponds to the reports of Sendi and Salehi (2010) on *Papilio demoleus* L (Lep: Papilionidae) exerted by an IGR methoprene. Similarly, Rahimi et al. (2013) and Khosravi et al. (2014) also showed decreased number of cells in their respective insects upon IGRs treatment. On the effects of plant extracts or essential oils on THC, there are plenty of available literature (Sharma et al., 2003; Ghasemi et al., 2014; Shaurub et al., 2014; Dhivya et al., 2018; Sadeghi et al., 2019). The DHC in spite of increasing trend, the corresponding pictures, are clearly indicative of sever damages exerted by plant extract. Although, the cells are recognizable in their respective forms should not be considered as true and active hemocytes. The increase in DHC particularly the immunocytes (Grs and Pls) have been reported by Shaurub and Sabbour (2017) with *Melia azedarach* (Meliaceae) fruit extract on *Agrotis ipsilon* Hufnagel (Lep: Noctuidae) and others (Sharma et al., 2008). The increase in DHC is somehow confusing, since the observation as live (Phase contrast) or permanent slides stained with Geimsa showing damaging cells but not degenerated after 24–48 h. Our results in damaged cells are corresponding to the reports of some other workers in their respective studies (Altuntas et al., 2012; Sadeghi et al., 2019).

Reproduction in insects has been the target for pest control and thus chemicals that could suppress insect reproduction has attracted scientists‘ attention long time ago. Hence, this aspect of pest control via biorational chemicals have been the core of several researches (Sahayaraj and Alakiaraj, 2006; Riddiford, 2012; Ribeiro et al., 2015; Lau et al., 2018; Abdelgaleil et al., 2019; Couto et al., 2019). In the present study, the extract used also suppressed *G. pyloalis* fecundity which was complemented with histological and biochemical investigation on the adult ovary resulting from treated larvae. Costa et al. (2004) and Milano et al. (2010) reported suppression in fecundity and egg viability by essential oils. Engelman (1998) believes that even ovariol number is affected following insufficient feeding at younger stages and also exposed to secondary metabolites during its ovarial development. Birah et al. (2010) and Alves et al. (2014) working on clove and long pepper extract, observed a juvenoid like effect which altered *S. litura* and *Spodoptera frugiperda* Smith (Lep: Noctuidae) reproduction, respectively. Clove oil in their study reduced number of eggs and their viability has also been reported by Cruz et al. (2016) in *S. frugipedra*. Our results are in agreement with the reported investigations mentioned, where reduced number of eggs laid and decreased amount of important compounds including, Protein, triglyceride and glycogen are corresponding to the defects in histology treated insects ovaries.

## Conclusion

Our results on the effect of *W. somnifera* seed extract on *G. pyloalis* larvae clearly supports earlier findings of the effect of this plant extract on various insects studied. We also found out that, the extract not only had toxicity, but also prolonged later life stages, thus showing effects similar to exogenous growth hormones. This is presumed by the intermediate types formed in later stages, leading to insect mortality. This study also throws some light on the mode of action of this toxic substance. This was evident in reduced activity of main digestive enzymes, macromolecules, and sever damage to cellular structure of the midgut. Another aspect of consideration in mode of action of this extract, was to study immunological aspect. Thus severe morphological damages were evident on these cells making them susceptible to diseases. We also noticed that those insects reaching adult stage were unable to produce eggs. The histology of ovary clearly depicted the loss of yolk, loosening of trophocytes and formation of vacuoles in the trohocytes and oocytes. Vitillin and chorion were not formed in the terminal oocytes of treated insects complemented with reduced energy reserves. Therefore, the *Withania* extract has the potential to be considered for further studies and a candidate for non-chemical insect pest control.

## Data Availability Statement

The datasets generated for this study are available on request to the corresponding author.

## Author Contributions

JS and ZA conceived, designed, and performed the experiments and wrote the first draft of the manuscript. AK-M and AZ helped in analysis and edited the manuscript. All authors approved the final draft.

## Conflict of Interest

The authors declare that the research was conducted in the absence of any commercial or financial relationships that could be construed as a potential conflict of interest.
